# The National Wealth Score in the *Brazilian National Survey on Child Nutrition* (ENANI-2019)

**DOI:** 10.1590/0102-311XEN050822

**Published:** 2023-08-28

**Authors:** Pedro Gomes Andrade, Raquel Schincaglia, Dayana Rodrigues Farias, Inês Rugani Ribeiro de Castro, Luiz Antonio dos Anjos, Elisa Maria de Aquino Lacerda, Cristiano Siqueira Boccolini, Nadya Helena Alves-Santos, Paula Normando, Maiara Brusco de Freitas, Neilane Bertoni, Gilberto Kac, Brazilian Consortium on Child Nutrition

**Affiliations:** 1 Instituto de Nutrição Josué de Castro, Universidade Federal do Rio de Janeiro, Rio de Janeiro, Brasil.; 2 Instituto de Nutrição, Universidade do Estado do Rio de Janeiro, Rio de Janeiro, Brasil.; 3 Departamento de Nutrição Social, Universidade Federal Fluminense, Niterói, Brasil.; 4 Instituto de Comunicação e Informação Científica e Tecnológica em Saúde, Fundação Oswaldo Cruz, Rio de Janeiro, Brasil.; 5 Instituto de Estudos em Saúde e Biológicas, Universidade Federal do Sul e Sudeste do Pará, Marabá, Brasil.; 6 Divisão de Pesquisa Populacional, Instituto Nacional de Câncer José Alencar Gomes da Silva, Rio de Janeiro, Brasil.

**Keywords:** Population Characteristics, Socioeconomic Status, Surveys and Questionnaires, Principal Component Analysis, Características da População, Nível Socioeconômico, Inquéritos e Questionários, Análise de Componente Principal, Características de la Población, Nivel Socioeconómico, Encuestas y Cuestionarios, Análisis de Componente Principal

## Abstract

The National Wealth Score (IEN) is a synthetic household index that assesses socioeconomic conditions. This study aims to present the methods used to update the IEN using data from the *Brazilian National Survey on Child Nutrition* (ENANI-2019). The following items were included: the education level of the mother or caregiver of the child; the number of bedrooms and bathrooms, TV sets, and cars in the household; and the presence of a radio, refrigerator or freezer, washing machine, microwave oven, telephone line, computers, air conditioner, media player devices, cable or satellite TV, cell phone ownership and type of service, cell phone internet, and internet at the household. Principal component analysis (PCA) was used to estimate the IEN with and without incorporating the complex sampling design (CSD). Thus, the IEN validation considered proxy indicators of socioeconomic status and living conditions. The first component of the PCA explained 31% and 71% of the variation with and without incorporating the CSD, respectively. The coefficients of variation of the IEN were 53.4% and 2.6% with and without incorporating the CSD, respectively. The mean IEN score was lower in households without access to a sewage system, those that received benefits from Brazilian Income Transfer Program, those with some degree of food insecurity, and those with stunted children. Adding ENANI-2019 items to the calculation of IEN to capture technological advances resulted in a better fit of the model. Incorporating the CSD increased PCA performance and the IEN precision. The new IEN has an adequate performance in determining the socioeconomic status of households with children aged under five years.

## Abstract (pt)

O Indicador Econômico Nacional (IEN) é um índice domiciliar sintético que avalia condições socioeconômicas. Este estudo tem como objetivo apresentar os métodos utilizados para atualização do IEN a partir de dados do *Estudo Nacional de Alimentação e Nutrição Infantil* (ENANI-2019). Foram incluídos os seguintes itens: escolaridade da mãe/cuidador da criança; o número de quartos e banheiros, aparelhos de TV e carros no domicílio; a presença de rádio, geladeira ou freezer, máquina de lavar, forno micro-ondas, linha telefônica, computadores, ar-condicionado, aparelhos multimídia, TV a cabo ou via satélite, propriedade e tipo de serviço de telefone celular, rede de dados de telefone celular e internet no domicílio. A análise de componentes principais (ACP) foi utilizada para estimar o IEN com e sem a incorporação da amostragem complexa. Assim, a validação do IEN considerou indicadores *proxy* de nível socioeconômico e condições de vida. O primeiro componente da ACP explicou 31% e 71% da variação com e sem a incorporação da amostragem complexa, respectivamente. Os coeficientes de variação do IEN foram de 53,4% e 2,6% com e sem a incorporação da amostragem complexa, respectivamente. O escore médio do IEN foi menor em domicílios sem acesso a esgoto, naqueles que receberam benefícios do Programa Bolsa Família, naqueles com algum grau de insegurança alimentar e naqueles com crianças com déficit de crescimento. A adição de itens do ENANI-2019 ao cálculo do IEN, a fim de capturar os avanços tecnológicos, resultou em um melhor ajuste do modelo. A incorporação da amostragem complexa aumentou o desempenho da ACP e a precisão do IEN. O novo IEN tem um desempenho adequado na determinação do nível socioeconômico de domicílios com crianças menores de cinco anos.

## Abstract (es)

El Indicador Económico Nacional (IEN) es un índice domiciliar que evalúa las condiciones socioeconómicas. Este estudio tiene como objetivo presentar los métodos utilizados en la actualización del IEN con base en datos del *Estudio Nacional de Alimentación y Nutrición Infantil* (ENANI-2019). Se incluyeron los siguientes ítems: nivel educativo de la madre/cuidador del niño; la cantidad de dormitorios y baños, televisores y autos en el hogar; la tenencia de radio, heladera o freezer, lavadora, horno de microondas, línea telefónica, computadoras, aire acondicionado, equipo multimedia, televisión por cable o satélite, titularidad y tipo de servicio de telefonía celular, red de datos celular e internet en el hogar. Se utilizó el análisis de componentes principales (ACP) para estimar el IEN con y sin la incorporación de muestreo complejo. Así, la validación del IEN consideró indicadores *proxy* de nivel socioeconómico y condiciones de vida. El primer componente ACP explicó el 31% y el 71% de la variación con y sin la incorporación de muestreo complejo, respectivamente. Los coeficientes de variación del IEN fueron el 53,4% y el 2,6% con y sin incorporación de muestreo complejo, respectivamente. El puntaje medio del IEN fue menor en los hogares sin acceso a alcantarillado, en aquellos que recibieron beneficios del Programa Bolsa Família, en aquellos con algún grado de inseguridad alimentaria y en aquellos con niños con retraso en el crecimiento. La incorporación de los ítems del ENANI-2019 en el cálculo del IEN, con el fin de capturar los avances tecnológicos, dio como resultado un mejor ajuste del modelo. La incorporación de muestreo complejo incrementó el desempeño de la ACP y la precisión del IEN. El nuevo IEN tiene un desempeño adecuado para estimar el nivel socioeconómico de los hogares con niños menores de cinco años.

## Introduction

The accurate assessment of the economic condition of a household is a challenge faced by most household surveys, especially those not primarily designed to measure income and expenditure or social inequalities. To solve this problem, Barros & Victora ^1^ conceived the National Wealth Score (IEN), which is a synthetic household index using the 2000 *Brazilian Demographic Censu*s data. It was developed as an alternative to the direct use of household income and incorporates items related to the possession of consumer goods, household characteristics, and the education level of the head of the household.

The use of the IEN to stratify the socioeconomic status of households in population surveys presents several strengths since this index is based on non-monetary income and, thus, mitigates problems regarding the quality of the reported income data ^2^ ^,^ ^3^ ^,^ ^4^. Another advantage is that the IEN considers consumer goods and the education level of the head of the household ^5^. The IEN has been used in Brazilian epidemiological studies since its creation ^6^ ^,^ ^7^ ^,^ ^8^ ^,^ ^9^ ^,^ ^10^ ^,^ ^11^ ^,^ ^12^.

However, Ewerling & Barros ^12^ found that the original IEN equation quickly lost its discriminatory capacity between 2002 and 2014. Changes in technology, increases in education level, and changes in the consumption profile of the Brazilian population occurred during this period, making the update and re-estimation of this index a priority. Therefore, this study aims to present methods for updating the IEN using data from the *Brazilian National Survey on Child Nutrition* (ENANI-2019), a household survey conducted in a probability sample of Brazilian children < 5 years old in 2019 ^13^. Another aim was to analyze the effect of incorporating the complex sampling design of the ENANI-2019 on the IEN accuracy and validate it using other proxy indicators of socioeconomic and living conditions.

## Methods

### Data source and variables

The ENANI-2019 is a Brazilian national household survey that aims to guide the formulation and reorientation of public policies on food, nutrition, and the health of children aged under five years. The study is a rich source of socioeconomic information, especially non-monetary income, for Brazilian households with children under five. It was used as an essential reference to portray the socioeconomic status of Brazilian households with children in that age group in the pre-COVID-19 pandemic period. The ENANI-2019 surveyed a sample of 12,524 households with 14,558 children aged under five years. That study had a complex sampling design, with geographic stratification, clustering by census tracts, and weight calibration that allowed the generation of estimates for Brazil as a whole as well as different macroregion, sex, and age groups ^14^.

The items used to construct the IEN were based on the characteristics of the child’s caregiver and household information extracted from the ENANI-2019 questionnaire ^13^. To estimate the IEN, the following items originally proposed by Barros & Victora ^1^ were selected: the education level of the mother or caregiver; the number of bedrooms, bathrooms, TV sets, and cars; the presence of a radio, fridge or freezer, washing machine, microwave oven, telephone line, microcomputer, or air conditioner. Possession of a videocassette recorder (VCR), included in the original proposition, was replaced by two items: possession of a media player and possession of cable/satellite TV. In addition to the original list, the following items were included: cell phone ownership and type of cell phone service (prepaid recharge, postpaid plan), cell phone internet, and access to the internet. These modifications and additions sought to address the technological development since the 2000’s and represent changes in the Brazilian population’s consumption profile ^12^.

Less than 1% of the data for a few items used in estimating the IEN were imputed using the hot deck method, a suitable method for categorical data, to replace implausible values and answers options “do not know or do not want to answer”, as detailed in Alves-Santos et al. ^13^ and Vasconcellos et al. ^14^.

### Update and validation of IEN

The update of the IEN was performed using Principal Component Analysis (PCA). PCA allows for the identification of interdependence between a set of variables of interest and reduces the dimensionality to provide a smaller number of synthetic indices, or scores, for each of the components created ^15^. The first component estimated for the IEN was used since it captured the highest percentage of the total explained variance of the dataset. Thus, it was the most suitable component for determining the household economic condition. This procedure was previously adopted by Barros & Victora ^1^.

The selection of variables to include in the PCA was carried out after obtaining two values: the Kaiser-Meyer-Olkin (KMO) statistic and the measure sample adequacy (MSA). Both values assess the suitability of a dataset for PCA. The KMO is a global measure; the MSA measures each variable and helps to exclude variables that do not meet criteria ^16^. The R programming language and its specific packages were used for all analyses (https://www.r-project.org). These indicators were estimated using the correlation matrix (*svycor function*, *jtools package*), allowing incorporation of the complex sampling design. For comparison, the estimation was also performed disregarding the complex sampling design (cor function of the base R and the KMO function of the *psych package*) (https://cran.r-project.org/package=psych). Then, extraction of the components, estimation of the proportion of explained variance, and calculation of the IEN scores were carried out.

To estimate the IEN, it was necessary to recode the original variables collected in the field, transforming them into numerical variables and, when necessary, adding some response categories. For example, the variable number of bedrooms was coded from 1 to 4, corresponding to the answers 1, 2, 3, and 4 or more, respectively. In all cases, the resulting variables were ordinal.

The estimation of the IEN was performed using the unit of the household. However, in the ENANI-2019, the head of the household was not defined; instead, information was collected on the mother or caregiver of each child. Thus, in the case of households with more than one mother or caregiver, the one with the highest education level was selected. As a result, only one education level per household was considered. This procedure was performed in 147 of the 12,524 households studied.

The estimation of the synthetic indices that incorporate the sampling design of the obtained data in later analyses is relatively underexplored, but when analyzing data from surveys with a complex sampling design, studies should consider the incorporation of the sampling design to produce a more robust point and variance estimates ^17^ ^,^ ^18^. Thus, the estimation of the IEN from the ENANI-2019 data was carried out, incorporating the complex sampling design. However, for evaluation purposes, the IEN was also calculated without considering the complex sampling design. The *svyprcomp function* of the *survey package* was used to incorporate the complex sampling design, and the *prcomp function* of the base R was used for the calculation without the complex sampling design.

The evaluation of the effect of incorporating the complex sampling design in the IEN was carried out in three ways: using the coefficient of variation of the estimates (CV), i.e., a measure of dispersion that indicates the heterogeneity of the data, obtained by the ratio between the standard error and the estimated value of the indicator multiplied by 100 to estimate the percentage of variation, which allowed for the measurement of precision; using the amount of total variation explained; and according to the score distribution.

The validation of the IEN incorporating the complex sampling design was performed using two procedures. The first by examining the association of the IEN with the total household income using Spearman’s correlation analysis (*weightedCorr function* of the *wCorr package*). Respondents reported total household income, calculated as the sum of the monetary income of all household members, including job, retirement, pensions, government benefits, savings accounts, rent, and other financial investments. The total household income was chosen over per capita income, as the IEN is a measure that better discriminates the total income and is not adjusted for the number of people in the household ^1^. The analysis of total income was conducted on a natural logarithmic scale using Spearman’s correlation. This was the same method used by Barros & Victora ^1^, which allows comparison between the studies.

Then, the mean IEN for the various household and child categorical variables were analyzed, allowing the discrimination of socioeconomic status and living conditions, using graphs with 95% confidence intervals (95%CI) and the packages *survey*, *srvyr*, and *tidyverse* ^19^. The following variables were used: (i) access to sewage system (public sewage or rainwater drainage network); (ii) presence of a beneficiary of the Brazilian Income Transfer Program in the household; (iii) household classification on the *Brazilian Food Insecurity Scale* (EBIA) as secure or with light/moderate/severe insecurity ^20^ ^,^ ^21^; and (iv) the Z score of the height-for-age index, which was used to classify children into stunting (< -2) and adequate height (≥ -2) categories according to the World Health Organization (WHO) reference curve ^22^ ^,^ ^23^. Notably, this last variable used the child as the unit of analysis. These indicators were selected because they are essential in studies on wealth inequality and have been used or are proposed to be analyzed in the future to evaluate the performance of the IEN ^1^.

After validation, the average total household income was calculated for each IEN category. The categorization of the IEN was performed based on measures of position: thirds, fourths, fifths, and tenths; incorporating the complex sampling design (*survey_quantile function*, *srvyr package*) and plotted with 95%CI. The final IEN score was calculated incorporating the complex sampling design, and its category (a measure of position: thirds, quarters, fifths, and tenths) was determined for each child, according to the location of their residence.

### Ethical considerations

The ENANI-2019 was approved by the Research Ethics Committee of the Clementino Fraga Filho University Hospital of the Federal University of Rio de Janeiro (UFRJ; CAAE n. 89798718.7.0000.5257). Data were collected after a parent or caregiver of the child authorized participation in the study through informed consent form.

## Results

### IEN estimation

Regarding education level, 67.7% of mothers or caregivers had levels ranging from the 9th grade to incomplete higher education. A total of 25.3% households had one bedroom; 52.7% had two bedrooms. At least one car was reported in 43.2% of the households, and TV sets were reported in 87.4%. Only a small fraction of households did not have cell phones, and home internet was observed in 61.6% of households (Table 1).

**Table 1:** Household characteristics of the variables used to estimate the National Wealth Score (IEN). *Brazilian National Survey on Child Nutrition* (ENANI-2019).

Characteristics/Category	Sample	Frequency	Households *	CV **
n	%	x 1,000	%
Education level (mother or caregiver)				
Up to 4th grade	599	4.3	555.8	9.9
5th to 8th grade	2,842	17.0	2,178.5	4.2
9th and 11th grade	3,361	20.3	2,592.7	4.1
12th grade up to incomplete higher education	6,399	47.4	6,058.3	2.5
Complete higher education	1,357	11.0	1,401.3	7.5
Number of bedrooms				
1	3,789	25.3	3,239.2	6.0
2	7,534	52.7	6,739.8	2.2
3	2,741	18.6	2,374.2	5.4
≥ 4	494	3.4	433.4	13.0
Number of bathrooms				
None	152	1.1	139.1	31.6
1	11,805	80.4	10,281.2	1.4
2	2,185	15.0	1,912.7	7.0
≥ 3	416	3.5	453.7	11.3
Number of TV sets				
None	503	2.6	331.5	11.0
1	10,127	68.3	8,728.6	1.6
2	3,005	21.6	2,761	4.4
≥ 3	923	7.6	965.5	9.5
Number of cars				
None	8,595	56.8	7,259.6	2.8
1	5,220	36.8	4,703.3	3.4
2	666	5.7	728.9	13.2
≥ 3	77	0.7	94.8	23.2
Radio				
No	8,307	53.5	6,844.5	3.1
Yes	6,251	46.5	5,942.2	3.6
Fridge or freezer				
No	308	2.0	257.3	13.2
Yes	14,250	98.0	12,529.4	0.3
Media player device				
No	9,408	61.6	7,874.0	2.6
Yes	5,150	38.4	4,912.6	4.1
Washing machine				
No	5,297	36.3	4,640.8	3.3
Yes	9,261	63.7	8,145.8	1.9
Microwave oven				
No	7,530	50.0	6,391.5	4.4
Yes	7,028	50.0	6,395.2	4.4
Computer				
No	9,599	63.9	8,166.1	2.7
Yes	4,959	36.1	4,620.5	4.8
Air conditioner				
No	11,456	81.0	10,356.3	2.4
Yes	3,102	19.0	2,430.3	10.0
Cable or satellite TV				
No	10,746	71.7	9,162.1	2.0
Yes	3,812	28.3	3,624.5	5.1
Home phone				
No	12,458	84.6	10,814.8	1.2
Yes	2,100	15.4	1,971.8	6.5
Cell phone and type of service				
No	423	3.1	394.7	14.8
Prepaid	11,674	78.1	9,987.0	2.0
Postpaid	2,461	18.8	2,404.9	7.6
Internet on cell phone				
No	1,570	11.2	1,438.2	6.9
Yes	12,988	88.8	11,348.4	0.9
Internet at home				
No	6,063	38.4	4,904.8	4.8
Yes, only cable Internet	1,944	13.2	1,687.0	11.3
Yes, cable and wireless Internet	6,551	48.4	6,194.9	4.3

CV: coeficient of variantion.

* The cell values in the table must be multiplied by 1,000 to obtain the total number of households with children aged under 5 years in that condition;

** A measure of dispersion that indicates the data heterogeneity, obtained by the ratio between the standard error and the estimated mean value of the indicator, multiplied by 100.

Note: frequency, households, and CV were estimated incorporating the complex sampling design.

The MSA was greater than 0.73 for all variables, and the KMO was 0.89 with or without incorporating the complex sampling design. These results indicate that the set of variables was suitable for conducting a PCA and that none of them should be excluded from the analysis. The IEN score that incorporated the complex sampling design had negative loadings, indicating that the higher the IEN score, the worse the household socioeconomic status. To facilitate results interpretation, we multiplied the result by -1, meaning that the higher the IEN score, the better the socioeconomic status of the household. The IEN that incorporated the complex sampling design differed in the magnitude of the loadings and in the order of its contribution. The variation explained by the first component with the complex sampling design was more than twice the value of the first component without the complex sampling design (Table 2).

**Table 2:** Component loadings with and without incorporation of the complex sampling design in the estimation of the National Wealth Score (IEN). *Brazilian National Survey on Child Nutrition* (ENANI-2019).

Characteristic	IEN without incorporating the complex sampling design		IEN incorporating the complex sampling design	
	Loading	MSA	Loading	MSA
Education level (mother or caregiver)	0.59	0.90	-0.57	0.90
Number of bedrooms	0.26	0.81	-0.44	0.81
Number of bathrooms	0.18	0.86	-0.27	0.87
Number of TV sets	0.24	0.89	-0.31	0.89
Number of cars	0.28	0.92	-0.14	0.92
Radio	0.04	0.73	-0.11	0.76
Fridge or freezer	0.01	0.87	-0.19	0.87
Media player	0.08	0.84	-0.09	0.86
Washing machine	0.17	0.90	-0.15	0.90
Microwave oven	0.18	0.90	-0.12	0.91
Computer	0.21	0.91	-0.10	0.91
Air conditioner	0.11	0.88	-0.05	0.89
Cable or satellite TV	0.15	0.92	-0.08	0.92
Home phone	0.11	0.92	-0.05	0.92
Cell phone and type of service	0.12	0.86	-0.25	0.87
Internet on cell phone	0.06	0.78	-0.18	0.80
Internet at home	0.49	0.91	-0.29	0.90
KMO	0.89		0.89	
Percentage of the variation explained by the 1st component	31.25		71.07	

KMO: Kaiser-Meyer-Olkin; MSA: measure of sampling adequacy.

**Figure 1:**
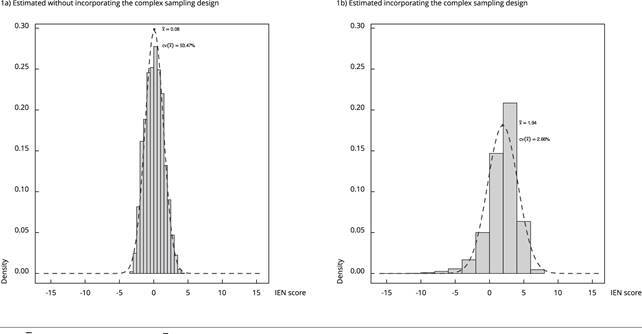
Distribution of the National Wealth Score (IEN) estimated with and without incorporating the complex sampling design. *Brazilian National Survey on Child Nutrition* (ENANI-2019).

Note: x̅ is the estimated mean, and CV(x̅) is the coefficient of variation of the mean.

The mean CV of the IEN calculated with the complex sampling design was 2.66% and the one calculated without the complex sampling design was 53.47%. When the complex sampling design was incorporated into the analysis, a greater representation in the tails of the distribution of the population was observed (Figure 1). Households in the Southeast Region of Brazil were concentrated in the highest fifths of the IEN distribution, indicating that the residents of this region have a higher socioeconomic status than those in other regions (Table 3). In contrast, households in the North and Central-West regions were concentrated in the lowest fifths of the IEN distribution. This unbalanced distribution indicates significant socioeconomic heterogeneity in Brazil and that wealth is concentrated in the Southeast Region.

**Table 3:** Distribution (%) of Brazilian households with children aged under 5 years in each quintile of the National Wealth Score (IEN), estimated incorporating the complex sampling design by macroregion. *Brazilian National Survey on Child Nutrition* (ENANI-2019).

Macroregion	IEN quintile				
	1st	2nd	3rd	4th	5th
North	34.73	21.78	19.77	14.66	9.05
Northeast	16.18	22.64	21.98	22.69	16.50
Southeast	10.28	14.00	19.60	24.09	32.03
South	22.73	26.09	21.64	16.21	13.33
Central-West	54.68	26.23	12.05	5.37	1.66

Note: 1st: lowest socioeconomic status; 5th: highest socioeconomic status. The estimate for Brazil overall was omitted, as it has a constant relative distribution of 20% every fifth.

### IEN validation

**Figure 2:**
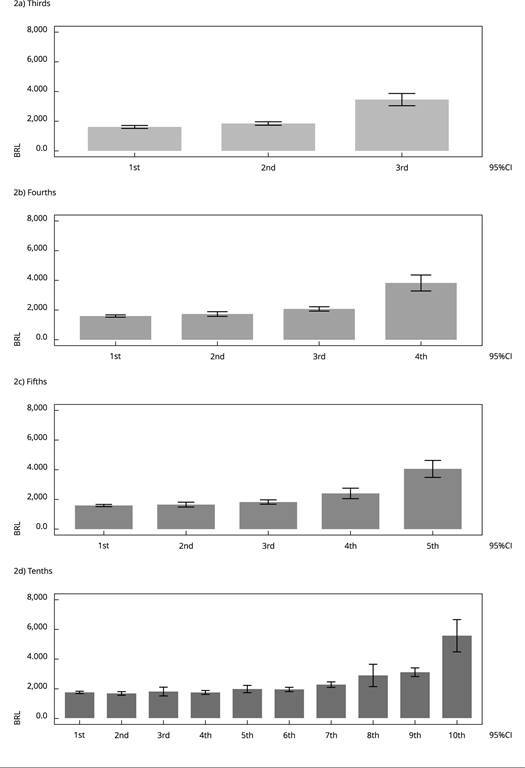
The mean and 95% confidence interval (95%CI) of total monthly household income (Brazilian Reais, BRL) according to various distributions of the National Wealth Score (IEN) estimated incorporating the complex sampling design. *Brazilian National Survey on Child Nutrition* (ENANI-2019).

Note: 1st: lowest socioeconomic status; last: highest socioeconomic status.

A gradient of mean total household income was observed and the IEN score could be stratified into thirds, fourths, fifths, and tenths, indicating that the higher ranges of the indicator had higher incomes (Figure 2). By examining the 95%CI, we observed that the greater the number of groups in the stratified IEN score, the more similar the average earnings observed in the groups with the lowest socioeconomic status were. The Spearman’s correlation between the IEN score incorporating the complex sampling design and total household income was 0.39.

**Figure 3:**
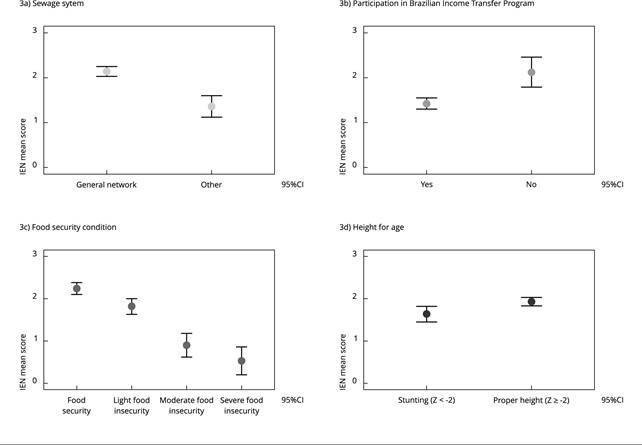
The mean and 95% confidence interval (95%CI) of the National Wealth Score (IEN) according to the presence of household sewage system, participation in Brazilian Income Transfer Program, food insecurity situation of the household, and child height. *Brazilian National Survey on Child Nutrition* (ENANI-2019).

Note: height (presence or absence of stunting) according to Z scores of the height for age. The height analysis used the child as the unit of analysis.

The mean IEN score was significantly lower in households without access to a sewerage, residents enrolled in the Brazilian Income Transfer Program and those with some degree of food insecurity (Figure 3). Lower mean IEN scores were observed among children with stunted growth than in those with adequate height (Figure 3).

## Discussion

This study shows that updating the items that compose the IEN and incorporating the survey complex sampling design improved the discriminatory power and accuracy of the indicator. The updated IEN has improved as demonstrated by its validity, compared to the original version. The mean score of the indicator showed significant differences concerning the *proxy* item categories of socioeconomic status and living conditions.

Notably, the original proposal of the IEN considered only urban areas. However, given that the ENANI-2019 data includes less than 3% of the sampled households in rural areas, we decided to estimate the IEN for the entire sample. IEN originally uses the education level of the head of the household; however, in ENANI-2019, it was not possible to do so, and we used data on mother or caregivers. Unfortunately, it was not possible to analyze the influence of this change on the results. The ENANI-2019 questionnaire addressing the IEN incorporated new items (last five items in Table 2) to adjust for the technological advances that society has experienced since the 2000s. The MSA (> 0.7) and KMO (> 0.6) indicated that, currently, less important variables from the set of durable household goods, such as having a radio, are still essential for determining the socioeconomic status of households, justifying their inclusion in the current IEN calculation.

The estimated component loadings for the IEN, based on the ENANI-2019, indicated that education level was the variable with the highest loading. In contrast, Barros & Victora ^1^ found that the number of TV sets was the variable with the highest loading, and education was only the 8th variable among the 13 employed according to the 2000 *Brazilian Demographic Census* data. This result reflects the expansion of education level and the promotion of access to higher education by public policies implemented in this period ^24^, reducing the importance of the number of TV sets and increasing the importance of education level. The number of televisions continued to have a substantial weight in the estimation of the IEN, which presented a higher loading than access to the internet at home and having a cell phone.

The incorporation of the complex sampling design in the PCA increased the discriminatory power of the indicator. A higher percentage of explained variation, an improvement in the mean accuracy of the IEN score, and a greater spread of IEN scores were observed compared to analyses without the complex sampling design. These findings indicate that the calculation of the IEN, which incorporated the complex sampling design, was more robust and accurate. Other authors obtained similar results using simulations ^25^ ^,^ ^26^. However, incorporating the complex sampling design inverted the sign of the component loadings, indicating differences in polarity between the measurement methods. The measurement that did not incorporate the complex sampling design had a direct relationship with socioeconomic status (the higher the IEN score, the greater the socioeconomic status of the household); that is, it served as a wealth indicator. The opposite was observed when the analysis incorporated the complex sampling design, that is, the IEN was an indicator of poverty. To facilitate results interpretation, the score was multiplied by -1, making it an indicator of wealth, similar to the methods of other studies ^27^.

Barros & Victora ^1^ found that the first component explained 38% of the total variation in the 2000 *Brazilian Demographic Census* data. In the results of the IEN calculation from the ENANI-2019, data that did not incorporate the complex sampling design explained the same amount of the total variation. However, this value exceeded 70% when the complex sampling design was incorporated into the analysis. In PCAs, a component that explains more than 70% of the variation indicates that using only one component is enough to summarize the dataset ^15^.

The IEN validation process consisted of analyzing the relationship of its score, obtained using a complex sampling design, and indicators of socioeconomic status and living conditions, thus evaluating whether the IEN was an essential summary indicator of the socioeconomic status of Brazilian households. The correlation between total household income and the IEN score in this analysis was much weaker (0.39) than that observed by Barros & Victora ^1^ (0.74), suggesting that the IEN is currently less strongly associated with income. This result has several potential explanations, including the accuracy of monetary income, measurement quality ^2^ ^,^ ^3^ ^,^ ^4^, and factors such as loss of purchasing power during economic crisis (the circumstance in which the survey was conducted), which directly influence monetary income and, to a lesser extent, non-monetary income. Moreover, income can be seasonal and may show great variability, especially during crisis and in poorer families, an issue that do not tend to influence the permanent income and, consequently, the IEN score. Another explanation is that ENANI-2019 included only Brazilian households with children aged under five years, whereas the IEN original formulation was intended for all Brazilian households. Notably, using income to validate the IEN may not be the best strategy, as the IEN itself is based on the premise that income is not a good item for summarizing the socioeconomic status of households. However, analyzing the average income is the most intuitive way to present the stratification of the IEN scores.

Using a synthetic index to measure socioeconomic conditions, such as the IEN, is very useful in population surveys with only a few items to assess income, mainly because the IEN refers to non-monetary income, which is generally more accurately reported. The main challenges in these surveys include defining the level of detail for income assessment, the possibility of capturing seasonal income, specifying the reference period, determining monetary and/or non-monetary, and household or individual income, and dealing with the quality of self-reports, such as relying on memory or proxy informants ^2^ ^,^ ^3^ ^,^ ^4^. Furthermore, income has a high non-response rate, especially in individuals with higher incomes ^28^.

A single measure that synthesizes a set of elements that express the socioeconomic status of households is less preferable than a group of indicators, which allows the assessment of multiple indicators. Furthermore, summarizing information reduces the variability inherent to the phenomenon studied and can eliminate residual differences. Thus, the updated IEN is a satisfactory means of expressing the socioeconomic status of Brazilian households with children < 5 years old and an important mechanism for assessing socioeconomic stratification in the country.

In conclusion, the IEN is an indicator that expresses the socioeconomic status of households and has been constructed through items that can be easily implemented in epidemiological surveys. According to our results, the addition of new items to those of the index’s original items (1) captured technological advances; (2) resulted in a better quality of fit for the model; and (3) maintained its good performance in discriminating the socioeconomic status of Brazilian households with children < 5 years old, even nearly 20 years after its original formulation. Thus, the IEN is an alternative to assessing direct household income, as self-reports of direct household income are often lower in quality. The incorporation of the complex sampling design was fundamental in increasing the PCA’s performance and the indicator’s precision.
